# Experimental and Numerical Investigation of Dustfall Effect on Remote Sensing Retrieval Accuracy of Chlorophyll Content †

**DOI:** 10.3390/s19245530

**Published:** 2019-12-14

**Authors:** Baodong Ma, Xuexin Li, Aiman Liang, Yuteng Chen, Defu Che

**Affiliations:** 1 Key Laboratory of Ministry of Education on Safe Mining of Deep Metal Mines, Northeastern University, Shenyang 110819, China; 1701015@stu.neu.edu.cn (X.L.); 1800993@stu.neu.edu.cn (A.L.); chenyuteng001@163.com (Y.C.); chedefu@mail.neu.edu.cn (D.C.); 2 Institute for Geoinformatics & Digital Mine Research, Northeastern University, Shenyang 110819, China

**Keywords:** dustfall, chlorophyll content, hyperspectral vegetation index, retrieval accuracy, PROSPECT-based mixture model

## Abstract

Chlorophyll is the dominant pigment in the photosynthetic light-harvesting complexes that is related to the physiological function of leaves and is responsible for light absorption and energy transfer. Dust pollution has become an environmental problem in many areas in China, indicating that accurately estimating chlorophyll content of vegetation using remote sensing for assessing the vegetation growth status in dusty areas is vital. However, dust deposited on the leaf may affect the chlorophyll content retrieval accuracy. Thus, quantitatively studying the dustfall effect is essential. Using selected vegetation indices (VIs), the medium resolution imaging spectrometer terrestrial chlorophyll index (MTCI), and the double difference index (DD), we studied the retrieval accuracy of chlorophyll content at the leaf scale under dusty environments based on a laboratory experiment and spectra simulation. First, the retrieval accuracy under different dustfall amounts was studied based on a laboratory experiment. Then, the relationship between dustfall amount and fractional dustfall cover (FDC) was experimentally analyzed for spectra simulation of dusty leaves. Based on spectral data simulated using a PROSPECT-based mixture model, the sensitivity of VIs to dust under different chlorophyll contents was analyzed comprehensively, and the MTCI was modified to reduce its sensitivity to dust. The results showed that (1) according to experimental investigation, the DD model provides low retrieval accuracy, the MTCI model is highly accurate when the dustfall amount is less than 80 g/m^2^, and the retrieval accuracy decreases significantly when the dustfall amount is more than 80 g/m^2^; (2) a logarithmic relationship exists between FDC and dustfall amount, and the PROSPECT-based mixture model can simulate the leaf spectra under different dustfall amounts and different chlorophyll contents with a root mean square error of 0.015; and (3) according to numerical investigation, MTCI’s sensitivity to dust in the chlorophyll content range of 25 to 60 μg/cm^2^ is lower than in other chlorophyll content ranges; DD’s sensitivity to dust was generally high throughout the whole chlorophyll content range. These findings may contribute to quantitatively understanding the dustfall effect on the retrieval of chlorophyll content and would help to accurately retrieve chlorophyll content in dusty areas using remote sensing.

## 1. Introduction

**Figure 1:**
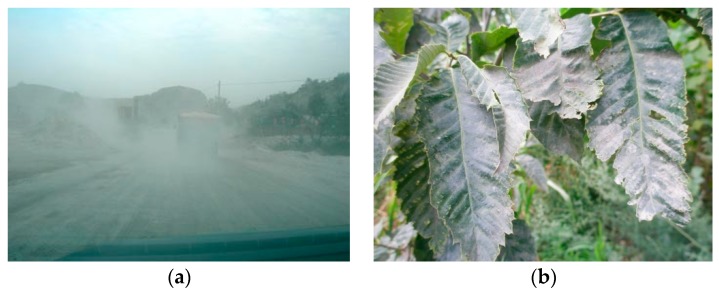
Dust pollution in an iron mining area in Northeast China: (**a**) dust dispersion and (**b**) dusty leaves around the mining area.

Dust is largely released by industrial and mining enterprises [1,2,3], becoming one of the main pollutants that affect air quality [4,5]. Dust aerosols can affect the climate and biogeochemistry [6,7], and pose a risk to human health [8]. In China, dust pollution is serious in some mining areas [9] for two reasons (Figure 1). Firstly, the concentration ration (the number of tons of ore required to produce one ton of concentrate) is high in some mining areas [10]. Thus, a large amount of ore is mined and transported to processing plants by truck, and large amounts of tailings are produced. As a result, massive amounts of dust pollution are produced. Secondly, some industrial and mining enterprises have not implemented effective dust prevention measures due to poor management. Therefore, large amounts of dust diffuse into the atmosphere in these areas. Anshan, located in Liaoning Province, northeastern China, is one of the largest iron and steel bases in China [11]. With increasing iron ore mining and iron tailings emission, dust pollution is serious in this area. After spreading a certain distance, part of the dust is deposited on the land surface (e.g., on native vegetation and crop plants) [12,13,14].

Vegetation is one of the best indicators for reflecting the quality of the regional ecosystem [15,16]. Vegetation responds to many environmental factors; thus, vegetation monitoring is important for understanding ecological construction, environment regulation, and grain yield. Remote sensing technology can be used to retrieve different physiochemical parameters, such as biomass and yield estimation [17], water content [18], vegetation type [19], leaf area index [20], and various pigments [21]. Chlorophyll is the dominant pigment in plant photosynthesis, and can reflect plant photosynthetic ability, physiological stress, carbon fixation capacity, and nitrogen use efficiency [22]. Therefore, chlorophyll content is an important index for evaluating the growth of vegetation, and it is widely used for vegetation monitoring [23]. Changes in chlorophyll content affect the spectra of vegetation, which enables the estimation of chlorophyll content using remote sensing data. In visible wavelengths, chlorophyll absorbs strongly in red and blue spectral regions, with maximum absorbance between 660 and 680 nm and maximum reflectance around 560 nm [24]. The hyperspectral vegetation index (VI), which is calculated based on the spectra, can be used to effectively retrieve the chlorophyll content of vegetation [25]. Various VIs have been developed to estimate chlorophyll content of vegetation at leaf and canopy scales [26]. Empirical methods are some of the widely used methods to retrieve chlorophyll content based on the relationship between chlorophyll content and VIs [27].

Accurate vegetation reflectance is critical for reliably estimating vegetation parameters based on empirical methods. However, sometimes an accurate reflectance of the target cannot be obtained due to the spectral masking caused by dust or weathering residues on the target [28]. This problem has drawn geologists’ attention [29]. Since rock surfaces are usually covered by weathered coating, spectral signatures of rocks would not be clean, but would include a spectral mixture of the two endmembers [30]. Similarly, dust falling on the leaf would change the spectral characteristics of vegetation [10,31]. According to the relationship between spectra and dusty leaves, reflectance spectroscopy could be used as an effective tool for monitoring soot pollution in urban suburbs [32]. Spectra-based VIs change with the dust amount [28]. Based on this, foliar dustfall amount was estimated by establishing models using multivariate linear regression, principal component regression, and partial least squares regression [33,34]. Dust falling on the leaf may reduce the retrieval accuracy of plant parameters when using empirical methods, but this has rarely been the focus of research, particularly in mining areas. The influence of foliar dust on nitrogen monitoring of crops beside a road was studied using reflectance spectra [35]. The authors found that foliar dust increases the nitrogen prediction error. The effect of dust on spectra-based estimation of leaf water content was also studied in Shanghai, China [36], where the accuracy of water content retrieval varied with dust amount. These studies were instructive for research on the retrieval of chlorophyll content in dusty environments. Until now, research mainly focused on the dust problem in urban areas but not mining areas. However, the dust amount is location-dependent and the amount of dust in urban areas is relatively low compared to mining areas [37]. The dustfall amount is usually less than 24 g/m^2^ in urban areas [38] but can be more than 110 g/m^2^ in mining areas [39]. Thus, studying the change in retrieval accuracy under the high dustfall amounts in mining areas is necessary.

In these studies on dusty leaves, the leaf samples were usually classified into two types: clean or dusty. This does not meet the needs of quantitative analysis under dusty conditions. From the perspective of chlorophyll content, retrieval accuracy may vary with chlorophyll content due to the different sensitivities of VIs at different chlorophyll contents [24,40]. Therefore, we considered a relatively wide leaf chlorophyll content range in our study. To quantitatively study the spectral change and its effect on retrieval accuracy of chlorophyll content, we first obtained the spectra of dusty leaves with different dustfall amounts and different chlorophyll contents. The spectra can be obtained by laboratory measurement [41]. However, obtaining leaf samples with continuous chlorophyll contents is difficult. Therefore, models were used to simulate the spectra of dusty leaves under different dustfall amounts and different chlorophyll contents [42]. The PROSPECT model, a simple but effective radiative transfer model, has been widely used to simulate leaf spectra for various purposes [43]. Thus, the PROSPECT model was selected to simulate the reflection spectra of leaves in dusty conditions.

The purpose of this study was to analyze the effect of dustfall on chlorophyll content retrieval using both experimental and numerical methods at the leaf scale. The findings lay the foundation for improving the chlorophyll content retrieval accuracy using hyperspectral remote sensing in dusty environments.

## 2. Materials and Methods

**Figure 2:**
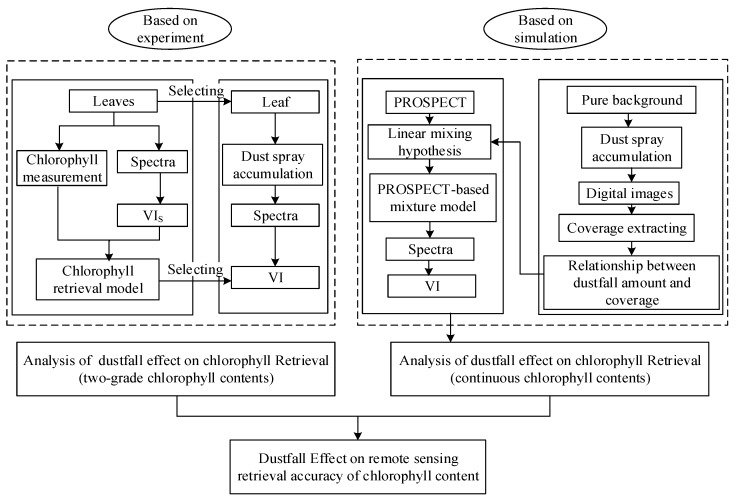
Flowchart of the study of dustfall effect on remote sensing chlorophyll content retrieval accuracy on the leaf scale. The experiment was conducted in the laboratory using manual dust spraying. The simulation method was based on the relationship between dustfall amount and coverage and spectra mixture to obtain the spectra under continuous chlorophyll contents. Abbreviations: VI: vegetation index.

We investigated the dustfall effect on the retrieval of chlorophyll content using an experiment and a simulation. Firstly, the dustfall effect was studied using the measured spectra of dusty leaves in an experiment. Since the experiment only provided a finite number of levels of chlorophyll content, a simulation method based on the dustfall amount–coverage relationship and spectra mixture was used to obtain the spectra under continuous chlorophyll contents. The flowchart of the method is shown in Figure 2.

### 2.1. Samples Collection and Spectra Measurement

Boston ivy leaf in the Anshan area was selected as the leaf sample for the experiment. The sample was fixed on the horizontal experimental platform immediately after being picked. Chlorophyll content was measured using SPAD-502 (Minolta Camera, Osaka, Japan). Five to seven points were measured evenly on each dust-free leaf, and the mean value SPAD was set as the chlorophyll content of the leaf. 

**Figure 3:**
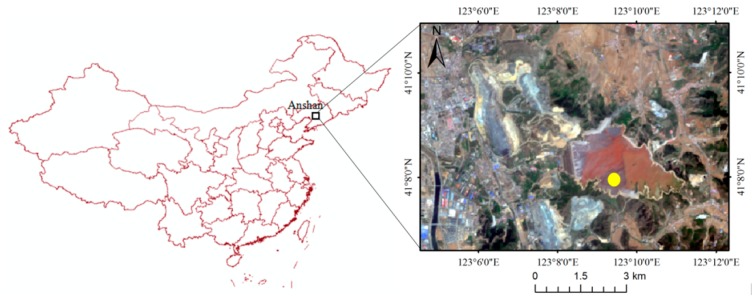
Location of the Qidashan tailings pond in Landsat 5 Thematic Mapper (TM) image (yellow point is the dust sample collection location).

The dustfall sample was collected from the Qidashan tailings pond, which is located in the east of Anshan, China (Figure 3). Its main elements include 82.28% SiO_2_, 9.90% TFe, 1.62% FeO, 0.85% MgO, 0.73% Al_2_O_3_, and 0.66% CaO. The particle size in the dustfall sample was less than 100 μm. 

**Figure 4:**
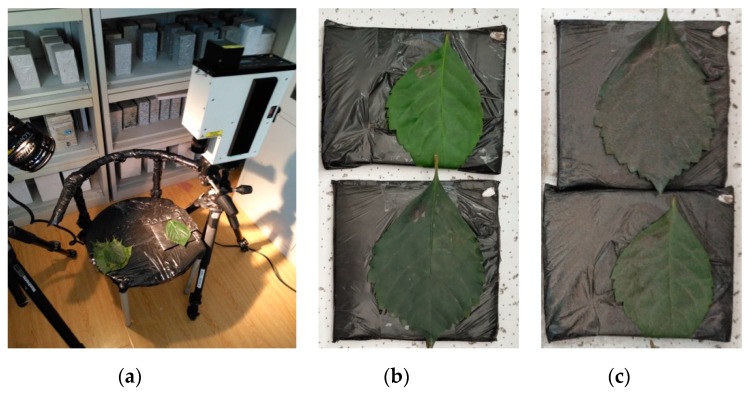
Spectra measurement of dust-free and dusty leaves in the experiment: (**a**) spectra measurement scene using SVC HR-1024 spectrometer, (**b**) dust-free leaf sample, and (**c**) dusty leaf sample.

In this experiment, SVC HR-1024 spectrometer (Spectra Vista Corporation, Poughkeepsie, NY, USA) was used to collect the spectra data of the leaves (Figure 4). The spectral resolution of the SVC HR-1024 spectrometer was 3.5 nm in the range of 350 to 1000 nm, 9.5 nm in the range of 1000 to 1850 nm, and 6.5 nm in the range of 1850 to 2500 nm. The experimental light source was a halogen lamp with 60° elevation angle and 50 cm distance from the target. Firstly, the spectral curves of 32 dust-free leave samples were measured to establish an empirical model for chlorophyll retrieval under dust-free conditions. Then, 2 of the 32 leaves (No. 1 with SPAD 50.9, and No. 2 with SPAD 65.2) were selected for the manual deposition of dust. Then, dust was sprayed on the two leaves with a difference. In the experiment, the dust was applied by air-blowing from eight different directions above the leaf sample with a constant height. We applied 20 different dustfall amounts from 8 to 160 g/m^2^ in 8 g/m^2^ increments. After the application was completed, a total of 21 sets of spectral curves were obtained for each leaf using an SVC HR-1024 spectrometer. Then the curves were merged and smoothed using the SVC HR-1024 PC Data Acquisition Software (Spectra Vista Corporation, Poughkeepsie, NY, USA).

### 2.2. Determining Dustfall Effect on Chlorophyll Content Retrieval Accuracy Based on Experiment

In this study, we established an empirical model to retrieve leaf chlorophyll content in a dust-free environment. The correlation between chlorophyll content and VIs of 32 leaves was analyzed to select the best retrieval model. The regression model was established and its determination coefficient (*R*^2^) was calculated using Excel (Microsoft Corporation, Redmond, WA, USA). The medium resolution imaging spectrometer terrestrial chlorophyll index (MTCI) [44] logarithmic model, and the double difference index (DD) [26] exponential model were selected for the next step due to their high correlation with chlorophyll content (Table 1 and Table 2). 

In this study, we used the relative error (RE) as the indicator of the retrieval accuracy of chlorophyll content:

(1) $$\mathrm{RE}=\frac{\left|y_{i}-y_{0}\right|}{y_{0}}$$

where *y_i_* is the predictive value of chlorophyll content according to the above retrieval model, and *y*_0_ is the measured value of chlorophyll content. The smaller the RE value, the higher the retrieval accuracy. According to the measured dusty leaf spectra, the RE of retrieval was calculated for each selected VI to determine the dustfall effect on chlorophyll content retrieval accuracy.

### 2.3. Relationship between Dustfall Coverage and Dustfall Amount for Dusty Leaf Spectral Simulation

The spectra of dusty leaves are a mixture of dust and leaf spectra. Thus, the fractional dustfall cover (FDC) of the dusty leaf, which describes the fractional abundance of a dusty leaf, is the key parameter used to determine mixed spectra. However, dustfall is usually measured as dustfall amount (weight per unit area) in the environmental field. Therefore, we needed to determine the relationship between FDC and dustfall amount prior to spectra simulation by directly using dustfall amount.

**Figure 5:**
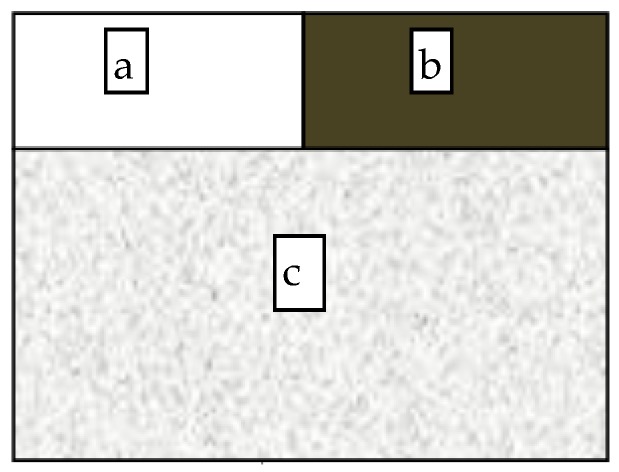
Experiment design for determining the relationship between dust amount and dust coverage: (**a**) dust-free background, (**b**) pure dust, and (**c**) dusty area.

The relationship was determined by designing an experiment. Dust was sprayed on the white background in intervals of 10 g/m^2^. An image was captured using a camera after each step (Figure 5). MATLAB (MathWorks, Natick, MA, USA) was used to process these images and calculate the FDC using the grayscale difference. The relationship of grayscale amongst the dust-free background, pure dust, and dusty area was determined based on a linear mixing model [45]:

(2) $$G_{1}f_{D}+G_{2}\left(1-f_{D}\right)=G_{3}$$

where *G*_1_ is the gray value of pure dust, *G*_2_ is the gray value of the dust-free background, *G*_3_ is the gray value of the dusty area, and *f_D_* is the FDC. The gray values of *G*_1_, *G*_2_, and *G*_3_ were obtained by processing the image acquired in the experiment. Thus, *f_D_* was calculated as:

(3) $$f_{D}=\frac{G_{3}-G_{2}}{G_{1}-G_{2}}$$

According to the calculated FDC and the known dustfall amount, the relationship between FDC and dustfall amount was determined. 

### 2.4. Quantitative Change Analysis of VIs for Different Dustfall and Chlorophyll Levels Based on Spectral Simulation

Manual collection of spectra is not only time-consuming and laborious, but also susceptible to experimental conditions. Numerical simulations can overcome these shortcomings and provide more eligible spectra. In this study, we considered the reflectance of dusty leaves as a linear superposition of the reflectance of pure dust and pure leaf. The reflectance of dusty leaves can be expressed as:

(4) $$R=R_{D}\times f_{D}+R_{L}\times \left(1-f_{D}\right)$$

where R is the reflectance of the dusty leaf, *R_D_* is the reflectance of dust, and *R_L_* is the reflectance of the leaf. *R_D_* was a constant because the dust sample is provided, *R_L_* was simulated by the PROSPECT model, and *f_D_* was calculated according to the relationship between FDC and dustfall amount when the dustfall amount is known. In other words, the spectra of dusty leaves with different dustfall amounts were simulated based on the PROSPECT model. As such, the above formula was called the PROSPECT-based mixture model. Given the ability to simulate spectra with different chlorophyll contents in PROSPECT, the model can simulate the spectra of dusty leaves with different chlorophyll contents and different dustfall amounts.

The chlorophyll content of the leaf samples in the dust-deposition experiment was within the range of 40 to 65 SPAD. According to the relationship between the SPAD value and actual chlorophyll content (μg/cm^2^), the 40–65 SPAD chlorophyll content on common vegetation leaves is equivalent to 40–90 μg/cm^2^ [46,47]. The PROSPECT-based mixture model was used to simulate the spectra of dusty leaves with chlorophyll content of 10–100 μg/cm^2^ in intervals of 5 μg/cm^2^. The spectra of dust leaves corresponding to each chlorophyll content value were set as one group, and the dustfall amount of each group was set to 0–160 g/m^2^ with an interval of 10 g/m^2^. A total of 19 groups of simulated leaf spectra were obtained with different chlorophyll contents, in which each group had 17 spectral curves with different levels of dustfall amounts. Root mean square error (RMSE) is the most widely used accuracy evaluation index as it reflects the average difference between the simulated and the measured values. Its calculation formula is as follows:

(5) $$RMSE=\sqrt{\frac{\sum_{j=1}^{n}{\left(y^{\prime }-y\right)}^{2}}{n}}$$

where *y′* is the simulated reflectance of the dusty leaf, *y* is the measured reflectance of the dusty leaf, and *n* represents the total number of actual bands in the spectra.

After obtaining the simulated spectra, these VIs were calculated, and their variation characteristics were analyzed under different chlorophyll contents and dustfall amounts. We comprehensively analyzed the dustfall effect on chlorophyll retrieval. 

## 3. Results

According to the experiment and simulation, spectra were obtained to study the effect of dustfall on the retrieval accuracy of chlorophyll content. The simulation-induced result provides a guideline for the retrieval of chlorophyll content.

### 3.1. Dustfall Effect on Chlorophyll Content Retrieval Accuracy Based on Experiment

**Figure 6:**
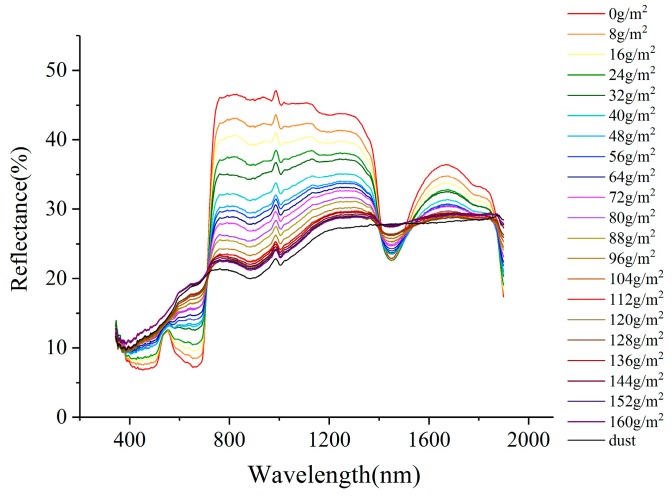
Leaf reflectance spectra change with dustfall amount.

According to the experiment results, as dustfall amount increased, the spectral curves of dusty leaves became similar to that of dust. One of the two experimental leaves was taken as an example (Figure 6). The reflectance of the dusty leaf was greater than the dust-free leaf in 380–710 nm and 1420–1570 nm. As dustfall amount increased, the reflectance of dusty leaf also increased. The wave peak at 559 nm gradually flattened. In 710–1420 nm and 1570–1900 nm, reflectance of the dust-free leaf was greater than that of the dusty leaf, and the reflectance decreased with increasing dustfall amount.

**Figure 7:**
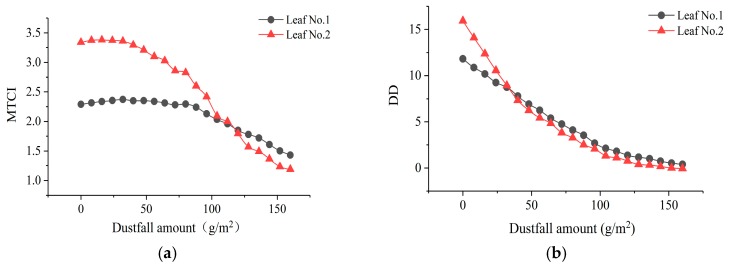
VI change with dustfall amount: (**a**) MTCI and (**b**) DD.

In general, the MTCI value was relatively stable when the dustfall amount was less than 80 g/m^2^, and the change rate increased when the dustfall amount was more than 80 g/m^2^ (Figure 7a). However, the change rate was different for the two leaves. The MTCI change rate of leaf No. 2 was greater than that of leaf No. 1. When dustfall amount was less than 100 g/m^2^, the DD value changed with increasing dustfall amount (Figure 7b). When the dustfall amount was greater than 100 g/m^2^, the DD value tended to stabilize.

**Figure 8:**
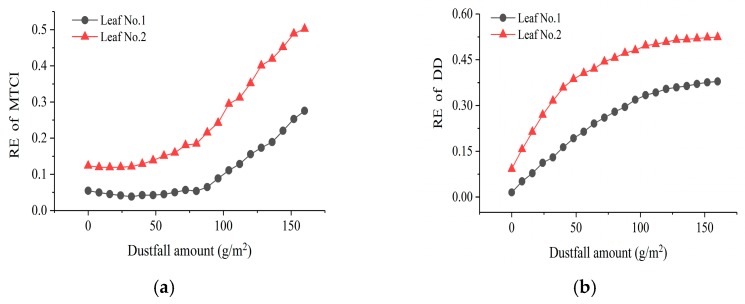
Retrieval accuracy varies with dustfall amount: (**a**) MTCI and (**b**) DD.

The chlorophyll content of dusty leaves was retrieved using MTCI and DD empirical retrieval models. The results showed that the MTCI model provided high retrieval accuracy when dustfall amount was less than 80 g/m^2^, but the retrieval accuracy decreased obviously when the dustfall amount was 80–160 g/m^2^ (Figure 8a). Conversely, with increasing dustfall amount, the retrieval accuracy of DD model decreased dramatically and then gently (Figure 8b).

### 3.2. Accuracy Analysis of Simulated Spectra by PROSPECT-Based Mixture Model

#### 3.2.1. Relationship between Dustfall Coverage and Dustfall Amount

**Figure 9:**
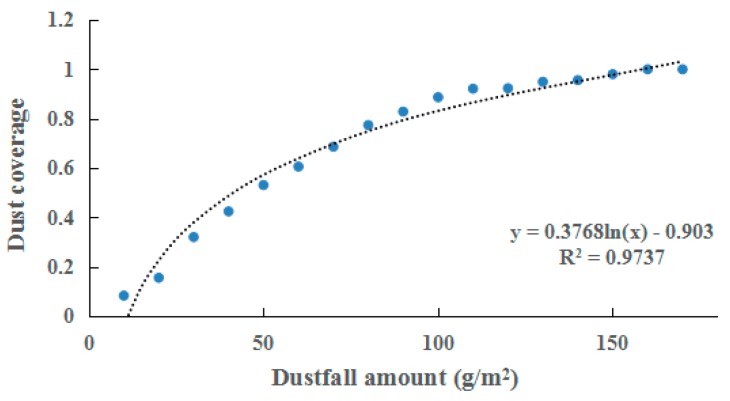
Relationship between fractional dustfall cover (FDC) and dustfall amount.

After the FDC was calculated by processing images acquired during the experiment, relationship between dustfall amount and FDC was described, as shown in Figure 9. We found a logarithmic relationship, and the determination coefficient (*R*^2^) was more than 0.97. FDC increased with increasing dustfall amount. However, the growth rate of FDC decreased gradually with the increasing dustfall amount when the dustfall amount was more than 80 g/m^2^.

#### 3.2.2. Accuracy Analysis of Simulated Spectra

**Figure 10:**
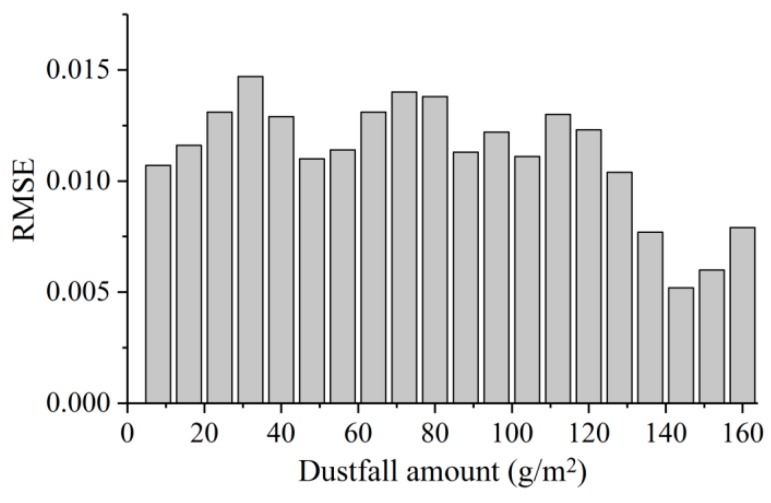
Root mean square error (RMSE) distribution of simulated spectra of dusty leaves under different dustfall amounts.

The spectra of dusty leaves were simulated using the PROSPECT-based mixture model. The maximum RMSE value was 0.015 when the dustfall was 32 g/m^2^ (Figure 10). RMSE declined overall when the dustfall amount was greater than 120 g/m^2^. According to the relationship between FDC and dust amount, FDC almost reached saturation when the dustfall amount was greater than 120 g/m^2^. Therefore, FDC deviation would not lead to large simulation errors in this range.

### 3.3. VIs Change Under Different Levels of Dustfall Amount and Chlorophyll Content Based on Simulation

The PROSPECT-based mixture model was used to simulate the spectral curves of dusty leaves for dustfall amounts of 0–160 g/m^2^ and chlorophyll contents of 10–100 μg/cm^2^. MTCI and DD were calculated for different chlorophyll contents and different dustfall amounts. In this study, change rate (*K*) of the VI under different dustfall amounts relative to the dust-free VI was defined to represent the sensitivity of the VI:

(6) $$K=\left|\frac{VI_{x}-VI_{0}}{VI_{0}}\right|$$

where *VI_x_* is the VI value when the dustfall amount is *x*, *VI*_0_ is the VI value of the dust-free leaf, and *K* is the change rate.

**Figure 11:**
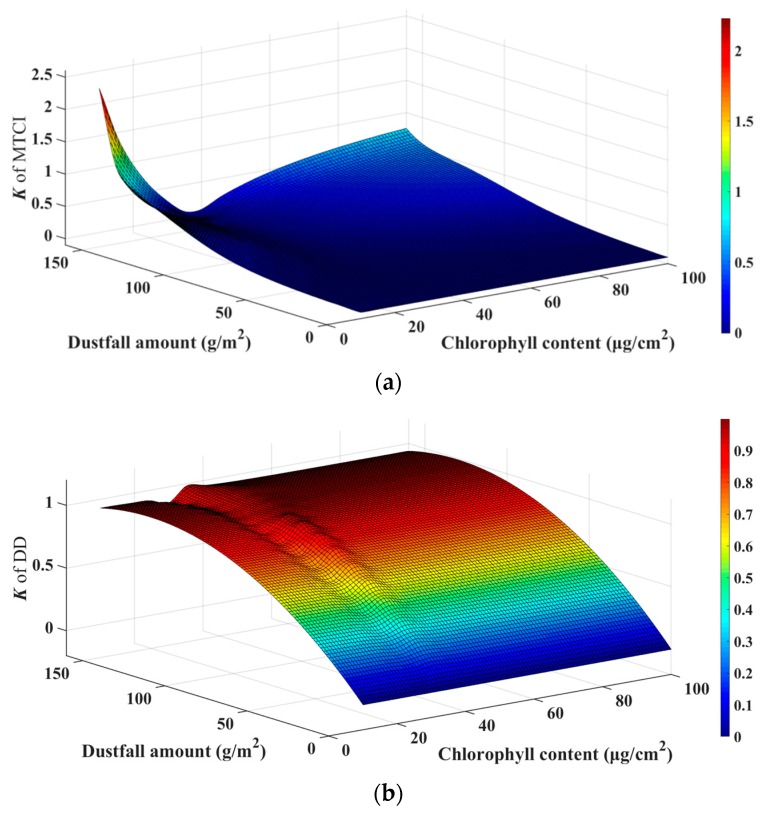
Relative rate of change of MTCI and DD for different chlorophyll contents: (**a**) MTCI and (**b**) DD.

Figure 11 shows that the sensitivity of MTCI and DD to dustfall varied significantly for different chlorophyll contents. For MTCI, *K* increased with increasing dustfall amount. When the dustfall amount was lower than 80 g/m^2^, *K* was relatively low. When the dustfall amount was greater than 80 g/m^2^, *K* increased significantly. For chlorophyll contents of 25–60 μg/cm^2^, *K* value was lower than 0.35. Outside this range, the *K* value changed considerably, with a maximum of 2.2. Therefore, MTCI showed good resistance to dust for chlorophyll contents of 25–60 μg/cm^2^ for the whole dustfall amount.

For DD, *K* also increased with increasing dustfall amount. However, the *K* value was relatively stable for different chlorophyll contents when dustfall amount was constant. The *K* value reached 0.4 when the dustfall amount was 40 g/m^2^ and was close to 1 when the dustfall amount was 160 g/m^2^. Therefore, DD is sensitive to dustfall amount for all chlorophyll content values, so it is not suitable for retrieving chlorophyll content under these conditions.

## 4. Discussion

### 4.1. Leaf Chlorophyll Content Retrieval Accuracy

In this study, we considered the spectra of dusty leaves as a linear mixture of dusty and dust-free leaves. However, the dust retained on the leaf may affect the spectra mixture nonlinearly [45]. For example, dust changes the roughness of the leaf surface. A shadow on the rough surface would affect the scattering of the electromagnetic wave when the incident angle is large [48]. In our laboratory experiment or spectra simulation, the incident angle was nearly vertical. Thus, the shadowing effect was ignored, although this was not possible in reality due to the off-vertical incident angle of the radiation source. We did not consider multiple reflection in the linear superposition. As a result, these factors may lead to errors during spectra simulation. In future studies, these factors should be considered to improve the accuracy of the PROSPECT-based mixture model.

The effect of dust deposition of leaf water content on estimation was studied in urban areas [36]. The results showed that the effect of dust cannot be ignored for remote sensing estimation of leaf water content. This is in agreement with the result of our study. However, the change in retrieval accuracy differs with increasing dust amount. The chlorophyll content retrieval accuracy decreased with increasing dust amount, but water content retrieval accuracy increased with increasing dust amount. This discrepancy may be related to the maximum dust amount and the retrieved parameter. The maximum dust amount considered was 5.53 g/m^2^ in the urban study and the maximum value considered was 160 g/m^2^ in our study. The large amount of dustfall in the study area occurs due to dust from the local mining, road surface conditions, and vehicle types. Due to the high dust amount, the difference between studied dust deposition amounts was 8 g/m^2^ in our experiment. At this level, a similar variation in retrieval accuracy was not detected. From the VI perspective, the differences in dust amount would also lead to differences in retrieval accuracy. For water content, the shortwave infrared water stress index (SIWSI) and water index (WI) have different retrieval accuracies [36], similar to MTCI and DD performing differently for chlorophyll content. Thus, we needed to test the performance of different VIs. From the perspective of plant parameters, the retrieval accuracy variation with the parameter should also be studied because the parameter usually varies widely in reality. However, this factor was not considered in previous studies on the dust effect [35,36]. In this study, MTCI provided a higher retrieval accuracy for chlorophyll contents of 25–60 μg/cm^2^ due to its low sensitivity in this range for the whole dustfall amount. This information could be useful for improving the applicability and practicability of the result.

The chlorophyll content retrieval accuracy was only studied on leaf scale in this research. Leaf scale is the basis of canopy scale. However, some factors should be considered when applying results from leaf scale to canopy scale. For example, leaf angle, leaf area, and height of canopy may affect the distribution of dust. Thus, these canopy factors should be considered for remote sensing applications in the future.

### 4.2. Attempt to Improve Retrieval Accuracy by Correcting MTCI

According to the results of the leaf dust deposition experiments, the retrieval accuracy of the DD model is low. The logarithmic MTCI model had a high retrieval accuracy when the dustfall amount was lower than 80 g/m^2^, which could be directly used to estimate chlorophyll content. When the dustfall amount was more than 80 g/m^2^, the MTCI retrieval accuracy was low with a certain regularity. MTCI was highly sensitive to chlorophyll content and could inhibit the influence of background and atmospheric changes [44]. Therefore, MTCI could be used for dust-effect modification to improve the accuracy of chlorophyll content retrieval under dustfall conditions. A multiplicative factor could be used to correct the MTCI value under dustfall conditions. The modified MTCI (mMTCI) is the product of MTCI and the multiplicative factor. The multiplicative factor was set as follows:

(7) $$\begin{array}{c} f_{x}=\frac{{\mathrm{MTCI}}_{0}}{{\mathrm{MTCI}}_{x}} \end{array}$$

where *f_x_* is the multiplicative factor, MTCI_0_ is the MTCI value of a dust-free leaf, and MTCI_x_ is the MTCI value when dustfall amount is *x*. Therefore, *f_x_* was obtained for different chlorophyll contents using the simulated spectra (Table 3).

**Figure 12:**
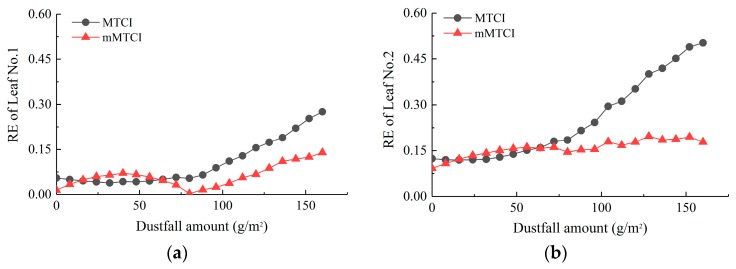
Accuracy improvement by modified MTCI (mMTCI) retrieval models: (**a**) leaf No. 1 and (**b**) leaf No. 2. RE, relative error.

We used the experimentally measured spectral data of dusty leaves to verify the correction result. The result showed that compared with the MTCI retrieval model, the dust sensitivity of the mMTCI retrieval model decreased and the retrieval accuracy improved significantly for dustfall amounts of 80–160 g/m^2^ (Figure 12). However, the multiplicative factor *f_x_* is dependent on chlorophyll content and dustfall amount, although the accuracy requirement for the chlorophyll content is not very high. The application of remote sensing in these scenarios would be hindered due to the difficulty of obtaining these prior parameters. Therefore, a more practical factor for modifying VIs for retrieval under dusty conditions should be studied in future research.

## 5. Conclusions

In this study, we examined the effect of dustfall on chlorophyll content retrieval. We first conducted a laboratory experiment to obtain the spectra of dusty leaves. The chlorophyll content retrieval accuracy for dusty leaves was preliminarily analyzed. Then, the spectra of dusty leaves were simulated under different chlorophyll contents and different dustfall amounts, and we analyzed the sensitivity of different VIs. The conclusions are summarized as follows:

(1) According to our experimental investigation, the DD model has low chlorophyll content retrieval accuracy for dusty leaves. The MTCI model produced high retrieval accuracy when the dustfall amount was lower than 80 g/m^2^, and its retrieval accuracy decreased significantly when the dustfall amount was greater than 80 g/m^2^. 

(2) We found a logarithmic relationship between fractional dustfall coverage (FDC) and dustfall amount. With increasing dustfall amount, dustfall coverage tended to be saturated when the dustfall amount was above 80 g/m^2^. On the basis of this relationship, the spectra of dusty leaves could be simulated using the PROSPECT-based mixture model, with an RMSE within 0.015.

(3) According to our numerical investigation, DD was highly sensitive to dust throughout the whole chlorophyll content range, and MTCI was less sensitive to dust for chlorophyll contents of 25–60 μg/cm^2^ than in other chlorophyll content ranges. MTCI could be used to retrieve the chlorophyll content of dusty leaves with modification.

Based on the laboratory experiment and simulation, we preliminarily explored the dustfall effect of iron tailings dust with hyperspectral retrieval of chlorophyll content, which could provide a reference for further research on the effect of dustfall on the retrieval of vegetation parameters. In practice, the relationship between FDC and dustfall amount and VIs’ sensitivity to dust may be related to dust characteristics. Therefore, a spectral database should be built containing typical dust in various mining areas. The related characteristics of dusty vegetation on leaf scale should be extended to canopy scale for remote sensing applications in future study.

## Figures and Tables

**Table 1:** Summary of vegetation indices (VIs).

	Formula	Reference
MTCI	(R749 – R709)/(R709 – R680)	[44]
DD	(R749 – R720) – (R700 – R671)	[26]

Abbreviations: MTCI: medium resolution imaging spectrometer terrestrial chlorophyll index; DD: double difference index.

**Table 2:** Retrieval model of leaf chlorophyll content (*n* = 32).

VI	Regression Equation	*R* ^2^
MTCI	*y* = 23.932lnx + 28.285	0.919 (*p* < 0.01)
DD	*y* = 31.095e^0.040*x*^	0.951 (*p* < 0.01)

**Table 3:** The results of multiplicative factor (*f_x_*) for MTCI correction for different chlorophyll contents.

Chlorophyll Content	Fitting Equation	*R* ^2^
60 μg/cm^2^	*f_x_* = 0.00006*x*^2^ – 0.0036*x* + 1.0421	0.981 (*p* < 0.01)
90 μg/cm^2^	*f_x_* = 0.0002*x*^2^ – 0.0112*x*+ 1.1798	0.982 (*p* < 0.01)

Note: *f_x_*_,_ the fitting equation for the multiplicative factor; *x* represents dustfall amount (g/m^2^).

## References

[1] Stachiw S., Bicalho B., Grant-Weaver I., Noernberg T., Shotyk W. (2019). Trace elements in berries collected near upgraders and open pit mines in the Athabasca Bituminous Sands Region (ABSR): Distinguishing atmospheric dust deposition from plant uptake. Sci. Total Environ..

[2] Zhang Z.H., Lin B.Q. (2019). Energy Conservation and Emission Reduction of Chinese Cement Industry: From a Perspective of Factor Substitutions. Emerg. Mark. Financ. Trade.

[3] Stovern M., Guzman H., Rine K.P., Felix O., King M., Ela W.P., Betterton E.A., Saez A.E. (2016). Windblown Dust Deposition Forecasting and Spread of Contamination around Mine Tailings. Atmosphere.

[4] Prospero J.M. (1999). Long-term measurements of the transport of African mineral dust to the southeastern United States: Implications for regional air quality. J. Geophys. Res. Atmos..

[5] de Longueville F., Hountondji Y.C., Ozer P., Marticorena B., Chatenet B., Henry S. (2013). Saharan Dust Impacts on Air Quality: What Are the Potential Health Risks in West Africa?. Hum. Ecol. Risk Assess..

[6] Mahowald N., Albani S., Kok J.F., Engelstaeder S., Scanza R., Ward D.S., Flanner M.G. (2014). The size distribution of desert dust aerosols and its impact on the Earth system. Aeolian Res..

[7] Miller R.L., Tegen I. (1998). Climate response to soil dust aerosols. J. Clim..

[8] Oduber F., Calvo A.I., Blanco-Alegre C., Castro A., Nunes T., Alves C., Sorribas M., Feraandez-Gonzalez D., Vega-Maray A.M., Valencia-Barrera R.M. (2019). Unusual winter Saharan dust intrusions at Northwest Spain: Air quality, radiative and health impacts. Sci. Total Environ..

[9] Li K.X., Liang T., Wang L.Q., Yang Z.P. (2015). Contamination and health risk assessment of heavy metals in road dust in Bayan Obo Mining Region in Inner Mongolia, North China. J. Geogr. Sci..

[10] Ma B.D., Pu R.L., Wu L.X., Zhang S. (2017). Vegetation Index Differencing for Estimating Foliar Dust in an Ultra-Low-Grade Magnetite Mining Area Using Landsat Imagery. IEEE Access.

[11] Zong Y.T., Xiao Q., Lu S.G. (2017). Magnetic signature and source identification of heavy metal contamination in urban soils of steel industrial city, Northeast China. J. Soils Sediments.

[12] Amato F., Pandolfi M., Viana M., Querol X., Alastuey A., Moreno T. (2009). Spatial and chemical patterns of PM10 in road dust deposited in urban environment. Atmos. Environ..

[13] Zender C.S., Bian H.S., Newman D. (2003). Mineral Dust Entrainment and Deposition (DEAD) model: Description and 1990s dust climatology. J. Geophys. Res. Atmos..

[14] Lovett G.M., Traynor M.M., Pouyat R.V., Carreiro M.M., Zhu W.X., Baxter J.W. (2000). Atmospheric deposition to oak forests along an urban-rural gradient. Environ. Sci. Technol..

[15] Imeson A.C., Prinsen H.A.M. (2004). Vegetation patterns as biological indicators for identifying runoff and sediment source and sink areas for semi-arid landscapes in Spain. Agric. Ecosyst. Environ..

[16] Kooistra L., Salas E.A.L., Clevers J., Wehrens R., Leuven R., Nienhuis P.H., Buydens L.M.C. (2004). Exploring field vegetation reflectance as an indicator of soil contamination in river floodplains. Environ. Pollut..

[17] Atzberger C. (2013). Advances in Remote Sensing of Agriculture: Context Description, Existing Operational Monitoring Systems and Major Information Needs. Remote Sens..

[18] Jackson T.J., Chen D.Y., Cosh M., Li F.Q., Anderson M., Walthall C., Doriaswamy P., Hunt E.R. (2004). Vegetation water content mapping using Landsat data derived normalized difference water index for corn and soybeans. Remote Sens. Environ..

[19] Xie Y.C., Sha Z.Y., Yu M. (2008). Remote sensing imagery in vegetation mapping: A review. J. Plant Ecol..

[20] Ren Z.B., Du Y.X., He X.Y., Pu R.L., Zheng H.F., Hu H.D. (2018). Spatiotemporal pattern of urban forest leaf area index in response to rapid urbanization and urban greening. J. For. Res..

[21] Kalacska M., Lalonde M., Moore T.R. (2015). Estimation of foliar chlorophyll and nitrogen content in an ombrotrophic bog from hyperspectral data: Scaling from leaf to image. Remote Sens. Environ..

[22] Garg H., Loughlin P.C., Willows R.D., Chen M. (2017). The C2(1)-formyl group in chlorophyll f originates from molecular oxygen. J. Biol. Chem..

[23] Vanbrabant Y., Tits L., Delalieux S., Pauly K., Verjans W., Somers B. (2019). Multitemporal Chlorophyll Mapping in Pome Fruit Orchards from Remotely Piloted Aircraft Systems. Remote Sens..

[24] Croft H., Chen J.M., Zhang Y. (2014). The applicability of empirical vegetation indices for determining leaf chlorophyll content over different leaf and canopy structures. Ecol. Complex..

[25] Heiskanen J., Rautiainen M., Stenberg P., Mottus M., Vesanto V.H. (2013). Sensitivity of narrowband vegetation indices to boreal forest LAI, reflectance seasonality and species composition. ISPRS J. Photogramm. Remote Sens..

[26] Le Maire G., Francois C., Dufrene E. (2004). Towards universal broad leaf chlorophyll indices using PROSPECT simulated database and hyperspectral reflectance measurements. Remote Sens. Environ..

[27] Zhang Y.Q., Chen J.M., Miller J.R., Noland T.L. (2008). Leaf chlorophyll content retrieval from airborne hyperspectral remote sensing imagery. Remote Sens. Environ..

[28] Ackerman D.E., Finlay J.C. (2019). Road dust biases NDVI and alters edaphic properties in Alaskan arctic tundra. Sci. Rep..

[29] Lyon R.J.P. Effects of weathering, desert-varnish, etc. on spectral signatures of mafic, ultramafic and felsic rocks, Leonora, West Australia. Proceedings of the 10th Annual International Symposium on Geoscience and Remote Sensing.

[30] Metelka V., Baratoux L., Jessell M.W., Naba S. (2015). Visible and infrared properties of unaltered to weathered rocks from Precambrian granite-greenstone terrains of the West African Craton. J. Afr. Earth Sci..

[31] Sun T.T., Lin W.P., Li Y., Guo P.P., Ying Z. (2017). Effect of Different Dust Weight Levels on Unban Canopy Reflectance Spectroscopy. Spectrosc. Spectr. Anal..

[32] Saaroni H., Chudnovsky A., Ben-Dor E. (2010). Reflectance spectroscopy is an effective tool for monitoring soot pollution in an urban suburb. Sci. Total Environ..

[33] Peng J., Xiang H.Y., Wang J.Q., Ji W.J., Liu W.Y., Chi C.M., Zuo T.G. (2013). Quantitative model of foliar dustfall content using hyperspectral remote sensing. J. Infrared Millim. Waves.

[34] Yan X., Shi W., Zhao W., Luo N. (2015). Mapping dustfall distribution in urban areas using remote sensing and ground spectral data. Sci. Total Environ..

[35] Wang T., Liu Y., Wu H.-Y., Zuo Y.-M. (2012). Influence of Foliar Dust on Crop Reflectance Spectrum and Nitrogen Monitoring. Spectrosc. Spectr. Anal..

[36] Lin W.P., Li Y., Du S.Q., Zheng Y.F., Gao J., Sun T.T. (2019). Effect of dust deposition on spectrum-based estimation of leaf water content in urban plant. Ecol. Indic..

[37] Brackx M., Wittenberghe S., Verhelst J., Scheunders P., Samson R. (2017). Hyperspectral leaf reflectance of *Carpinus betulus* L. saplings for urban air quality estimation. Environ. Pollut..

[38] Chen F.-T., Zhao W.-J., Yan X. (2015). The Study Based on Rectification of Vegetation Indices with Dust Impact. Spectrosc. Spectr. Anal..

[39] Zajec L., Gradinjan D., Klancnik K., Gaberscik A. (2016). Limestone dust alters the optical properties and traits of *Fagus sylvatica* leaves. Trees Struct. Funct..

[40] Datt B. (1998). Remote sensing of chlorophyll a, chlorophyll b, chlorophyll a + b, and total carotenoid content in eucalyptus leaves. Remote Sens. Environ..

[41] de Jong S.M., Addink E.A., Hoogenboom P., Nijland W. (2012). The spectral response of Buxus sempervirens to different types of environmental stress—A laboratory experiment. ISPRS J. Photogramm. Remote Sens..

[42] Chen H.Y., Huang W.J., Li W., Niu Z., Zhang L.M., Xing S.H. (2018). Estimation of LAI in Winter Wheat from Multi-Angular Hyperspectral VNIR Data: Effects of View Angles and Plant Architecture. Remote Sens..

[43] Jacquemoud S., Ustin S.L., Verdebout J., Schmuck G., Andreoli G., Hosgood B. (1996). Estimating leaf biochemistry using the PROSPECT leaf optical properties model. Remote Sens. Environ..

[44] Dash J., Curran P.J. (2004). The MERIS terrestrial chlorophyll index. Int. J. Remote Sens..

[45] Bioucas-Dias J.M., Plaza A., Dobigeon N., Parente M., Du Q., Gader P., Chanussot J. (2012). Hyperspectral Unmixing Overview: Geometrical, Statistical, and Sparse Regression-Based Approaches. IEEE J. Sel. Top. Appl. Earth Obs. Remote Sens..

[46] Uddling J., Gelang-Alfredsson J., Piikki K., Pleijel H. (2007). Evaluating the relationship between leaf chlorophyll concentration and SPAD-502 chlorophyll meter readings. Photosynth. Res..

[47] Yamamoto A., Nakamura T., Adu-Gyamfi J.J., Saigusa M. (2002). Relationship between chlorophyll content in leaves of sorghum and pigeonpea determined by extraction method and by chlorophyll meter (SPAD-502). J. Plant Nutr..

[48] Liu L., Mishchenko M.I. (2007). Scattering and radiative properties of complex soot and soot-containing aggregate particles. J. Quant. Spectrosc. Radiat. Transf..

